# Identification and validation of a novel prognostic model based on platinum resistance-related genes in bladder cancer

**DOI:** 10.1590/S1677-5538.IBJU.2022.0373

**Published:** 2022-11-20

**Authors:** Yining Hao, Chenghe Wang, Danfeng Xu

**Affiliations:** 1 Shanghai Jiao Tong University School of Medicine Ruijin Hospital Department of Urology Shanghai China Department of Urology, Ruijin Hospital, Shanghai Jiao Tong University School of Medicine, Shanghai, China

**Keywords:** Urinary Bladder Neoplasms, Chemotherapy, Adjuvant, Computational Biology

## Abstract

**Background:**

The depth of response to platinum in urothelial neoplasm tissues varies greatly. Biomarkers that have practical value in prognosis stratification are increasingly needed. Our study aimed to select a set of BC (bladder cancer)-related genes involved in both platinum resistance and survival, then use these genes to establish the prognostic model.

**Materials and Methods:**

Platinum resistance-related DEGs (differentially expressed genes) and tumorigenesis-related DEGs were identified. Ten most predictive co-DEGs were acquired followed by building a risk score model. Survival analysis and ROC (receiver operating characteristic) plot were used to evaluate the predictive accuracy. Combined with age and tumor stages, a nomogram was generated to create a graphical representation of survival rates at 1-, 3-, 5-, and 8-year in BC patients. The prognostic performance was validated in three independent BC datasets with platinum-based chemotherapy. The potential mechanism was explored by enrichment analysis.

**Results:**

PPP2R2B, TSPAN7, ATAD3C, SYT15, SAPCD1, AKR1B1, TCHH, AKAP12, AGLN3, and IGF2 were selected for our prognostic model. Patients in high- and low-risk groups exhibited a significant survival difference with HR (hazard ratio) = 2.7 (p < 0.0001). The prognostic nomogram of predicting 3-year OS (overall survival) for BC patients could yield an AUC (area under the curve) of 0.819. In the external validation dataset, the risk score also has a robust predictive ability.

**Conclusion:**

A prognostic model derived from platinum resistance-related genes was constructed, we confirmed its value in predicting platinum-based chemotherapy benefits and overall survival for BC patients. The model might assist in therapeutic decisions for bladder malignancy.

## INTRODUCTION

Bladder cancer (BC) is the 10^th^ most common cancer in the World (https://www.wcrf.org/cancer-trends/bladder-cancer-statistics), which carries a substantial social and financial burden. There were 573,278 new bladder cancer cases and 212,536 deaths worldwide in 2020 (1). Non-muscle-invasive bladder cancer (NMIBC) accounts for approximately 75% of BC patients, muscle-invasive bladder cancer (MIBC) accounts for 25% but has highly malignant potential and is closely related to mortality (3, 4). Platinum-based regimens have been the backbone treatment for MIBC patients in the first-line setting (5). Unfortunately, only half of patients were sensitive to these treatment (6), and a considerable proportion of patients who were initially sensitive to platinum will develop an acquired resistance during their treatment cycle, leading to a worse progression-free survival (PFS) or overall survival (OS) of patients with MIBC (7). Despite of similar clinicopathology features, the individual heterogeneity of genetics among malignancy cells brings significant differences in therapeutic response and outcomes, stressing the vital necessity for identifying platinum resistance biomarkers as well as the clinical route of BC.

Recent studies have discovered a series of biomarkers for platinum resistance, such as FOXC1 (8) and Circ_0058063 (9). Other evidence also showed the subtype of BC is associated with response to chemotherapy (10). These biomarkers are insufficient for effective treatment decisions because these studies were conducted on a molecular or cellular level and lacked prognostic information. With the advancements in transcriptomics, incorporating various biomarkers as well as clinical data to construct a risk stratification model has become a viable option (11). Compared to single biomarker, integrating multiple predictive genes into a single system would enhance the robustness and prognostic accuracy. Many gene signatures for prognosis in BC have sprung out recently. Wang et al. identified seven immune-related lncRNAs signature: Z84484.1, AC009120.2, AL450384.2, AC024060.1, TNFRSF14-AS1, AL354919.2, OCIAD1-AS1 (12). Yang et al. reported nine genes signature based on ferroptosis: ALB, BID, FADS2, FANCD2, IFNG, MIOX, PLIN4, SCD, and SLC2A3 (13). However, as far as we know, a platinum resistance-related model has not been reported before.

In this study, we aimed to identify essential bladder cancer-related genes involved in both platinum-based chemotherapy resistance and survival. Based on these genes, we established a risk score model and stratified patients into different risk groups. The robust prognostic ability of this model was verified in three independent BC datasets with platinum-based chemotherapy. Additionally, by integrating clinical features and risk score, a nomogram with enhanced prediction power was built. Besides, in an attempt to have a deeper understanding of this model, we used multiple databases to investigate the expression, functional interaction, and mutation of these genes. Enrichment analyses were carried out to further explore the possible mechanisms. As far as we know, this is the first prognostic model for predicting outcomes and discriminating responses to platinum-based chemotherapy in BC patients. Our model would play an important role in prognosis stratification and assisting individualized treatment.

## MATERIALS AND METHODS

### Data collection and preprocessing

**Figure 1:**
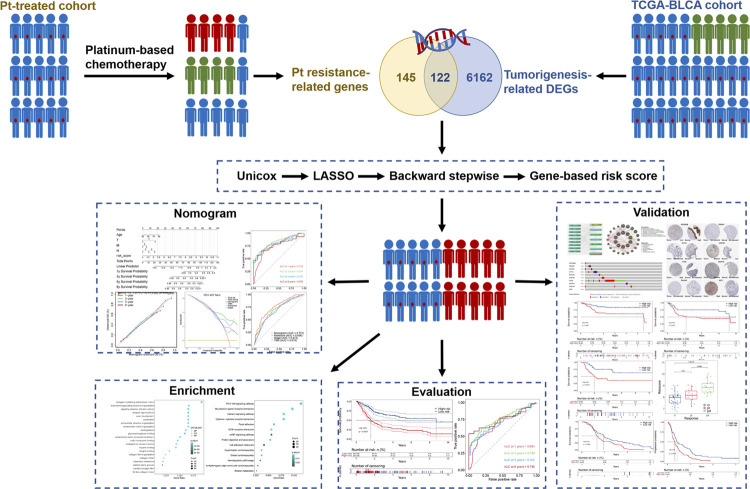
The overall design of the current study. Pt: Platinum.

The overall design of this study is shown in Figure-1. In training data, the RNA-seq data (Counts) with corresponding survival, phenotype, and clinical information of 411 BLCA (Bladder Urothelial Carcinoma) samples and 19 normal bladder tissues were collected from The Cancer Genome Atlas (TCGA) database (https://portal.gdc.cancer.gov/). Genes with low expression (the average expression < 1, or zero expression in more than 25% of samples) were excluded. Ensemble ID was converted to gene symbol by annotation file downloaded from the GENCODE website (https://www.gencodegenes.org/). In validation data, “bladder cancer” and “chemotherapy resistance” were used as the keywords for searching gene chips from the Gene Expression Omnibus (GEO) (https://www.ncbi.nlm.nih.gov/geo/). The inclusion criteria were as follows: (1) the biospecimens were gained from human primary bladder cells or tissues; (2) containing transcriptomic data; (3) including at least 10 samples in each group; (4) the survival information was available; (5) enrolling patients that had undergone platinum-based chemotherapy; (6) no previous or concomitant immunotherapy. Two independent GEO datasets (GSE13507 and GSE31684) (14–17) that meet our requirement were downloaded by “GEOquery” package (18). Entrez ID was converted to gene symbol according to platform files, only maximum expression was retained when multiple Entrez IDs were annotated by same gene symbol. Detailed information about three datasets is shown in Table-S1 (Click here). Patients with progressive disease were defined as platinum resistance, while patients with partial response and complete response were defined as platinum sensitive.

### Differentially expressed genes (DEGs)

The clinical information of patients enrolled was obtained by using “TCGAbiolinks” package (20–22), by focusing on those cases who undergone platinum-based chemotherapy, the acquired file contains 158 rows which were filtered to 100 unique samples, and 97 of them had corresponding gene expression data. The microarray data of samples with “Clinical Progressive Disease” and “Complete Response” therapeutic responses were imported into the “DESeq2” package (23), with a threshold of fold change > 2 or < 0.5, and adjust p value < 0.05, platinum resistance-related DEGs were defined. DEGs between primary tumor and normal samples were screened in the same way, meanwhile, the expression matrix was normalized, box plot proved batch effects among 430 samples were as well eliminated. Venn diagram depicting the intersection of platinum resistance-related DEGs and tumorigenesis-related DEGs in TCGA-BLCA dataset, those co-DEGs were retained for further Cox regression. Volcanic plots and heatmaps for DEGs were produced by “ggpubr” and “pheatmap” packages, respectively.

### A gene-based prognostic model

The expression data were log2 transformed to make the hazard ratio more significant. Of the 411 cases, 406 unique tumor biospecimens have associated survival information. Based on a criterion that the statistical significance threshold is log-rank P value < 0.05, a set of DEGs that were significantly related to prognosis were derived from univariate Cox regression. “Glmnet” package (24, 25) was used for least absolute shrinkage and selection operator (LASSO) Cox regression to subsequently select predictors, genes with non-zero coefficients were entered into backward stepwise regression. Finally, 10 optimal predictive genes and their coefficients were acquired, both Akaike's information criterion (AIC) value and the number of factors were all minimized. The risk score for each sample was calculated as follows:


$$\text{Risk score}=\sum_{i-1}^{n}\beta i*Expi.$$

where *βi* stants for regression coefficient of gene *i, Expi* stands for the expression level of gene i. Forest plot outlined hazard ratios (HR) and confidence intervals of 10 genes, survival map of them was plotted by the GEPIA 2 website (http://gepia2.cancer-pku.cn). Multicollinearity among them was tested by variation inflation factors (VIF) and correlation coefficients.

### Evaluating prognostic performance of gene-based model in training and validation groups

Univariable and multivariable Cox regression were performed to weigh up the predictive strength of risk score and other clinical parameters (age, gender, subtype, grade, stage, and TMN stage). Some characteristics which have very small numbers, for example, stage i, T0, and T1, were merged with their connected groups, and the results were summarized in forest plots. All patients in each dataset were classified into high-risk group and low-risk group, the cut-off points were based on median risk score (TCGA and GSE13507) or produced by X-tile software (GSE31684 and GSE14208). Survival risk differences between high- and low-risk groups were demonstrated by Kaplan-Meier survival analysis and log-rank test. Time-depended receiver operating characteristic (ROC) curves were applied to evaluate the prognostic performance of gene-based risk score with “TimeROC” package (26). Patients who have undergone platinum-based chemotherapy in TCGA and GSE13507 datasets were divided by their therapeutic responses, and boxplots were used for revealing the relationship between risk score and platinum resistance.

### Building and estimation of nomogram

Based on “rms” package, salient clinical parameters in multivariate Cox regression and risk score were enrolled into a nomogram model to predict 1-, 3-, 5-, and 8-year overall survival (OS) of BC patients. The discriminatory capacity of the nomogram model was estimated by ROC curve, meanwhile quantified by area under the curve (AUC) and concordance index (C-index). Sensitivity and specificity of different models were compared by “plotROC” package. Calibration plot revealed predictive accuracy of nomogram by comparing predicted survival rate with observed survival rate at different time points, the value of resampling was set to 1000 to reduce overfitting. Decision curve analysis (DCA) illustrated clinical net benefit of the nomogram model and other prognostic indicators with “dcurves” package, which proved the clinical utility of the nomogram.

### Functional enrichment and pathway analysis

We used “clusterProfiler” package (27) to reveal Gene Ontology (GO) and Kyoto Encyclopedia of Genes and Genomes (KEGG) pathways analysis for risk groups. P-values were adjusted by Benjamini and Hochberg's approach in order to check false discovery rate (FDR), enriched biological functions and activated pathways with FDR < 0.05 were picked out, and the top of them were exhibited in dot plots. Besides, we performed gene set enrichment analysis (GSEA) by GSEA_4.2.3 software with 1000 permutations. The BioCarta, KEGG, and CGP subsets of curated gene sets (C2), GO subset of ontology gene sets (C5), and oncogenic signature gene sets (C6) were downloaded from Molecular Signatures Database (MSigDB, http://www.gsea-msigdb.org/gsea/msigdb/index.jsp) (28–31). Significance criteria were nominal p value < 0.05, FDG < 0.25, and |NES| > 1.

### Co-occurrence analysis of chemotherapy resistance

COREMINE Medical website (http://www.coremine.com/medical) is a domain-specific information platform that mainly focused on biomedicine research and drug discovery. Employing text-mining, it allows users to navigate relationships among research contents from the latest published scientific literature. The keywords of “neoplasms”, “drug resistance”, and “cisplatin” were combined with 10 genes as inputs into the search field for co-occurrence analysis, then a graphic network of them was generated.

### Functional interaction network

GeneMANIA website (http://genemania.org) is a resource-rich tool for generating hypotheses about co-expression and functional interactions among genes (32). We imported the 10 genes in prognostic model into human database, 20 prioritized functionally similar genes were selected automatically, and a biological process weighted gene-gene network was constructed.

### Immunohistochemistry analysis

The Human Protein Atlas (THPA) (https://www.proteinatlas.org) is a database focusing on genome-wide analysis of human proteins, which contains expression data and immunohistochemically (IHC) stained tissue images of each protein-coding gene, establishing a correlation between tumor development and specific gene expression of 17 major cancer types. The expression levels of 10 genes, as well as the subcellular localization of their products between urothelial cancer and normal bladder, were compared by IHC images downloaded from “Pathology” and “Tissue” sections of THPA website, respectively. For same gene, in order to make the result more comparable, we chose images generated by identical antibody from similar patients. We also obtained 5-year survival rate of high expression group and low expression group regarding each protein to validate their impact on cancer patient survival.

### Gene mutation and copy number alteration

The cBioPortal (https://www.cbioportal.org) is a visualization tool for cancer genomics with large data sources (33, 34). It provides us an access to explore relationships between gene alteration and clinical features in specific cancer type or pan-cancer scope, and further explore the oncogenic mechanism at chromosomal level. We obtained the mutations and copy number alterations of 10 genes based on Bladder Cancer (MSK/TCGA, 2020) database, which incorporates 476 paired muscle-invasive bladder tumors and normal samples. The proportions of amplification, deep deletion, multiple alteration, and mutation were exhibited in stacked column charts.

### Quantification of immune cell infiltration

We applied CIBERSORTx (https://cibersortx.stanford.edu) to quantify the constitution of 22 human leukocyte types by using non-negative matrix factorization (NMF) algorithm (35, 36). The different infiltration degrees of immune cells in high-risk group and low-risk group were revealed in violin plot. Furthermore, for macrophages M0 and M2 that may have potential effect on anti-tumor treatment resistance, the correlation of risk score and their fractions were assessed by linear regression.

### Statistical analysis

Statistical analysis was carried out using R software (version 4.1.3; https://www.R-project.org/). Differences among GEO datasets and TCGA dataset were accessed by One-way Analysis of Variance (ANOVA) and Wald test, respectively. For continuous variables, differences between two groups were examined by Wilcoxon-Mann-Whitney (WMW) test or Student's t-test. For categorical variables, Chi-square test or Fisher's exact test were used to analyze assumptions depending on the proportion of groups that contain less than 5 patients. P-value < 0.05 was a statistical significance threshold for all analyses, with p < 0.1, *p < 0.05, **p < 0.01, ***p < 0.001.

## RESULTS

### Screening of platinum-based chemotherapy resistance-related genes

**Figure 2:**
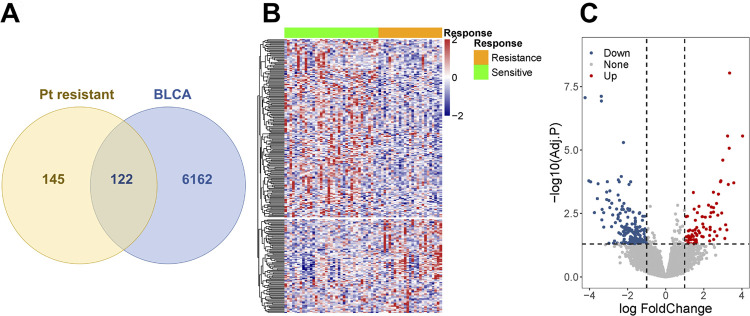
Differentially expressed genes of platinum-treated cohort in TCGA-BLCA dataset.

By using transcriptomic data of urothelium carcinoma samples that had undergone platinum-based chemotherapy in TCGA database, 267 DEGs between different therapeutic responses were confirmed, including 81 up-regulated and 186 down-regulated genes. Then, with all 430 samples in TCGA-BLCA database, 6284 DEGs were screened (Table-S2)(Click here). Venn diagram (Figure-2A) depicted 122 co-DEGs in these two cohorts. Heatmaps and volcano plots exhibited tumorigenesis-related (see appendix-1 - Figure-S1 A and B) and platinum-based chemotherapy resistance-related DEGs (Figure-2 B and C), respectively. Box plot (see appendix-1 Figure-S1 C) revealed the homogeneity of each sample after normalization.

### Identification of 10 genes with best predictive value

Thirty seven DEGs that were significantly associated with the overall survival of BC patients were selected by univariate Cox regression (p < 0.05, Table-S3) (Click here). LASSO Cox regression was used to reduce dimensions and prevent excessive fitting (see appendix-1 Figure-S2 A and B), and 21 DEGs were picked out (lambda = 0.018). After verifying the proportional hazard assumption, 10 genes with best predictive value were determined by backward stepwise regression: PPP2R2B, TSPAN7, ATAD3C, SYT15, SAPCD1, AKR1B1, TCHH, AKAP12, AGLN3, and IGF2. Hazard ratios (HRs) and confidence intervals of them are shown in forest plot (see appendix-1 Figure-S3).

**Figure 3:**
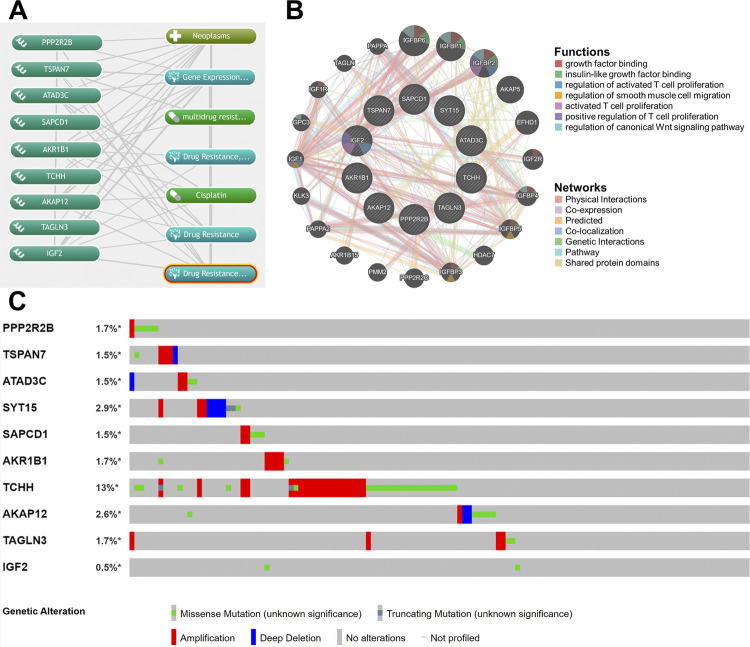
Co-occurrence, functional interaction, and copy number alteration of 10 genes.

We consulted the COREMINE Medical website about these genes. The results exhibited that, except for SYT15, the remaining 9 genes participate in oncogenicity and platinum-based chemotherapy resistance directly or indirectly (Figure-3A). Functional interaction, co-expression, biological process annotation, and gene-gene networks among these 10 genes were acquired from GeneMANIA website (Figure-3B). Additionally, Figure-3C elucidates the mutation type and ratio of each gene, 81 (18%) of 438 sequenced samples/patients have at least one alteration regarding 10 genes. To explore the gene expression on a protein level, we extracted immunohistochemistry (IHC) images of urothelial cancer and normal bladder samples regarding each gene (see appendix-1 Figure-S4). Among genes recorded in “Pathology” section in THPA, staining intensities of AKR1B1, TCHH, AKAP12, and IGF2 are distinctly higher in tissues from urothelial carcinoma than in normal bladder. PP2R2B and STY15 have evidently lower expression levels in bladder tumors versus non-cancerous tissues. Neither TSPAN7 nor TAGLN3 was detected by antibodies used in IHC staining. What is more, for gene that has elevated staining intensity in cancer cells, the 5-year survival rate of high expression group was worse compared with that of low expression group, and vice versa (Table-S4). (Click here). These all together were consistent with HRs of each gene in stepwise regression.

### A gene-based prognostic model

Through the above steps, we identified 10 platinum resistance-related genes with prognostic value in bladder urothelial carcinoma. In order to investigate the prognosis effect of 10 genes as a whole, we computed risk scores for every patient based on regression coefficients as follows:


$$\begin{array}{l} risk\;score=-0.354*Exp_{PPP2R2B}+0.770*Exp_{TSPAN7}\\ -0.6500*Exp_{ATAD3C}-0.451*Exp_{SYT15}-0.547\\ {}*Exp^{SAPCD1}+1.558*Exp_{AKRIBI}+0.468*Exp_{TCHH}\\ +0.459*Exp_{AKAPI2}+0.288*Exp_{TAGLN3}+0.644*Exp_{IGF2.} \end{array}$$

**Figure 4:**
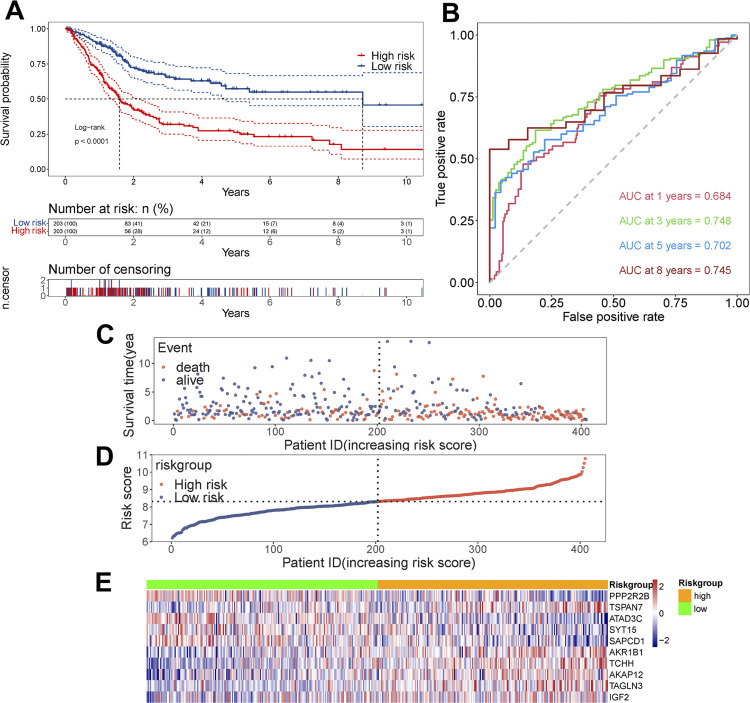
Evaluating prognostic performance of gene-based model in training group.

All patients in training dataset (TCGA-BLCA dataset) were divided into high-risk group and low-risk group based on the median risk score as critical value. Kaplan-Meier (K-M) plot showed a significantly enhanced overall survival of low-risk group than high-risk group (p < 0.0001; Figure-4A), the median survival time for low-risk group is 8.7 years and for high-risk group is 1.6 years. This gene-based risk score model yielded an area under the curve (AUC) of 0.684, 0.748, 0.702, and 0.745 in 1-, 3-, 5-, and 8-year survival prediction, respectively (Figure-4B), by its satisfactory predictive accuracy. Clinical features of patients were summarized in Table-1, the high-risk group was associated with non-papillary subtype, lower survival rate, shorter lifetime, and advanced disease (a higher stage, grade, and pathological TMN stages). In addition, we estimated the distribution of risk scores in OS and OS status. By listing patients in an order of increased risk scores (Figure-4C), we observed a worse survival in high-risk group (decreased in OS and increased number of deaths; Figure-4D). Heatmap of 10 genes displayed the trend of their expression levels with elevated risk scores (Figure-4E). For the purpose of exploring the intervention effects of different clinical characteristics and whether risk score works better for patients with certain conditions, we worked with subgroup analysis by breaking down all study samples into subsets based on age, gender, subtype, grade, stage, and pathological TMN stages. Prognosis of patients with high risk scores were poorer than patients with low risk scores in age < 68 (p < 0.0001), age ≥ 68 (p < 0.0001), female (p = 0.003), male (p < 0.0001), subtype of papillary (p = 0.00048), subtype of non-papillary (p < 0.0001), high grade (p < 0.0001), stage ii (p = 0.015), stage iii (p < 0.0001), stage iii+iv (p < 0.0001), stage iv (p = 0.017), pathological stage of T2 (p = 0.087), pathological stage of T3 (p < 0.0001), pathological stage of T3+T4 (p < 0.0001), pathological stage of T4 (p = 0.0036), pathological stage of M0 (p < 0.0001), pathological stage of Mx (p < 0.0001), pathological stage of N0 (p < 0.0001), and pathological stage of N1+N2+N3 (p = 0.037), the K-M plot and AUC of each subgroup were shown in appendix-1 Figure-S5.

**Table 1:** Clinical features of BC patients in TCGA dataset.

Clinical features		BC patients	Low-risk group	High-risk group	p-value
**Patients, no (%)**		406 (100)	203 (50)	203 (50)	
**Median age (years)**		68.5	66	70	
**Gender, no (%)**					0.572
	Female	106 (26.1)	50 (24.6)	56 (27.6)	
	Male	300 (73.9)	153 (75.4)	147 (72.4)	
**Stage, no (%)**					< 0.001
	Stage i + ii	131 (32.3)	84 (41.4)	47 (23.2)	
	Stage iii	141 (34.7)	68 (33.5)	73 (36.0)	
	Stage iv	132 (32.5)	49 (24.1)	83 (40.9)	
	Unknown	2 (0.5)	2 (1.0)	0 (0)	
**Grade, no (%)**					0.004
	Low grade	20 (4.9)	15 (7.4)	5 (2.5)	
	High grade	383 (94.3)	186 (91.6)	197 (97.0)	
	Unknown	3 (0.7)	2 (1.0)	1 (0.5)	
**Subtype, no (%)**					< 0.001
	Papillary	131 (32.3)	89 (43.8)	42 (20.7)	
	Non-Papillary	270 (66.5)	113 (55.7)	157 (77.3)	
	Unknown	5 (1.2)	1 (0.5)	4 (2.0)	
**Pathologic T, no (%)**					< 0.001
	T0 + T1 + T2	122 (30.0)	72 (35.5)	50 (24.6)	
	T3	193 (47.5)	81 (39.9)	112 (55.2)	
	T4	58 (14.3)	26 (12.8)	32 (15.8)	
	Unknown	33 (8.1)	24 (11.8)	9 (4.4)	
**Pathologic M, no (%)**					0.004
	M0	198 (48.8)	115 (56.7)	83 (40.9)	
	M1	11 (2.7)	3 (1.5)	8 (3.9)	
	Unknown	197 (48.5)	85 (41.9)	112 (55.2)	
**Pathologic N, no (%)**					0.003
	N0	236 (58.1)	126 (62.1)	110 (54.2)	
	N1	44 (10.8)	13 (6.4)	31 (15.3)	
	N2	76 (18.7)	33(16.3)	43 (21.2)	
	N3	7 (1.7)	2 (1.0)	5 (2.5)	
	Unknown	43 (10.6)	29 (14.3)	14 (6.9)	
**Status, no (%)**					< 0.001
	Alive	226 (55.7)	146 (71.9)	80 (39.4)	
	Dead	180 (44.3)	57 (28.1)	123 (60.6)	
**Median survival (days)**		536	603	455	

BC: Bladder cancer; TCGA: The Cancer Genome Atlas.

### Predictive ability of risk score in validation datasets

**Figure 5:**
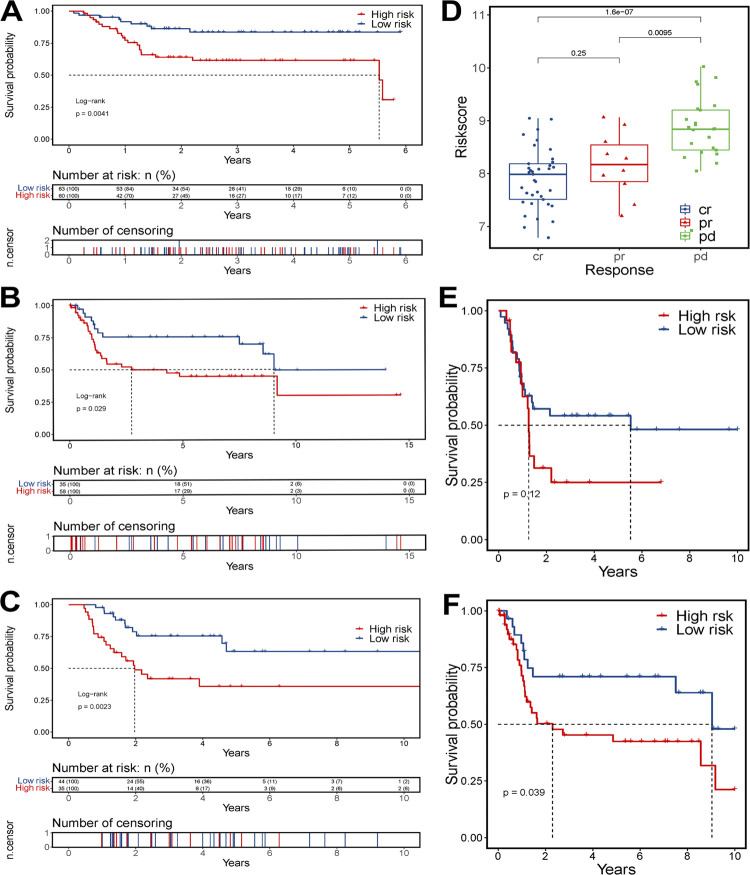
Evaluating prognostic performance of gene-based model in validation group.

The robust predictive ability of this gene-based risk score was validated in GSE13507 (Figure-5A), GSE31684 (Figure-5B), and a subset of patients who had undergone platinum-based chemotherapy in TCGA-BLCA dataset (Figure-5C). K-M plots of three databases showed that patients in low-risk group have better prognoses than patients in high-risk group (p = 0.0041, 0.029, and 0.0023, respectively). The time-dependent ROC curves possessed AUC values of 0.727 and 0.718 in predicting 3- and 5-year survival for GSE13507 (see appendix-1 Figure-S6A), 0.61 and 0.658 in predicting 3- and 8-year survival for GSE31684 (Figure-S6B), 0.841, 0.766, 0.707, 0.661 in predicting 1-, 3-, 5-, and 8-year survival for platinum-based chemotherapy-treated patients in TCGA-BLCA dataset (see appendix-1 Figure-S6C), respectively. Aiming to display the correlation between risk score and the sensitivity to platinum-based chemotherapy, we grouped platinum-treated patients in TCGA-BLCA dataset into three subsets according to their therapeutic response, risk scores of patients with “clinical progressive disease (pd)” after chemotherapy were significantly higher than patients in “partial response (pr)” group (p = 0.0095) and “completely response (cr)” group (p < 0.0001; Figure-5D). Then we used similar methods in GSE13507, patient who was sensitive to cisplatin generated a lower risk score than patient who was resistant to cisplatin (see appendix-1 Figure-S6D), suggesting that risk score is a predictor of decreased chemosensitivity. Furthermore, given that patients with less than T2 disease are not usually included in platin-based systemic treatment, the survival analyses were performed in patients from T2 or above T2 subgroups in GSE13507 (Figure-5E) and GSE31684 (Figure-5F). In these subgroups of two datasets, consistent with previous results, patients in low-risk group still had a better prognosis. The survival difference was not significant in the first year in GSE13507 (p=0.12), which may due to the interference of other comorbidities. The distribution of risk scores and survival status of GSE13507 was shown in (see appendix-1 Figure-S6E).

### Developing and evaluating the prediction nomogram

**Figure 6:**
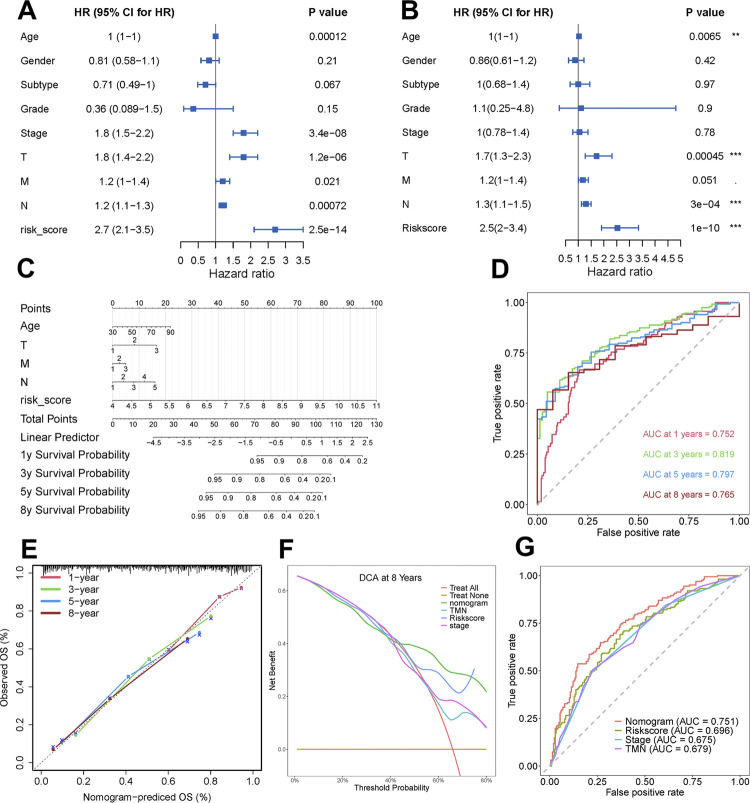
Building and estimation of nomogram.

Univariate Cox regression proved that age, stage, pathological T, M, N, and risk score were salient prognostic indicators (p < 0.05; Figure-6A), followed by multivariate Cox regression to test their independency (Figure-6B). Based on results of regressions, age, pathological T, M, and N were combined with gene-based risk score to develop a prediction model with nomogram, which was able to work out numerical probabilities of 1-, 3-, 5-, and 8-year overall survival (Figure-6C). The performance of nomogram was first examined by ROC analysis (Figure-6D). The concordance index (C-index) of nomogram was 0.727, and AUC of predicting 1-, 3-, 5-, and 8-year survival rates could reach 0.752, 0.819, 0.797, and 0.765, respectively. Then, in calibration plot, broken lines proximately coincide with the diagonal line, confirming the predictive accuracy of nomogram (Figure-6E). Decision curve analyses (DCA) in predicting 3- (see appendix-1 Figure-S7A), 5- (see appendix-1 Figure-S7B), and 8-year survival rates (Figure-6F) were conducted to determine whether a judgment method could enhance clinical decision at a specific threshold. The curve of nomogram (green) generated the greatest net benefit, followed by risk score, declaring their excellent clinical utility. Besides, this result also illustrated that the incorporation of gene-based risk score could enhance predictive performance. From the comparation of predictive abilities among nomogram, risk score, stage, and pathological TMN stages, both AUC (0.751; Figure-6G) and specificity (85.2%; Table-2) of nomogram outperformed other clinical characteristics, proving its prognostic value.

**Table 2:** Comparison of prediction performance among different models.

Models	AUC	95% CI	SE	Sensitivity (%)	Specificity (%)	p-value
Risk score	0.696	0.642-0.750	0.028	69.1	63.1	< 0.001
1-year	0.684	0.608-0.759	0.038			
3-year	0.748	0..685-0.812	0.032			
5-year	0.702	0.625-0.779	0.039			
8-year	0.745	0.651-0.838	0.048			
Nomogram	0.751	0.701-0.801	0.025	53.7	85.2	< 0.001
1-year	0.752	0.687-0.818	0.033			
3-year	0.819	0.764-0.873	0.028			
5-year	0.797	0.732-0.863	0.033			
8-year	0.765	0.671-0.859	0.048			
Stage	0.675	0.623-0.727	0.026	48.8	78.3	< 0.001
TMN	0.679	0.625-0.734	0.028	50.6	77.3	< 0.001

AUC = Area under the curve; CI = Confidence interval; SE = Standard error; TMN = The pathological T, M and N stages of tumor.

### GO, KEGG, and GSEA

**Figure 7:**
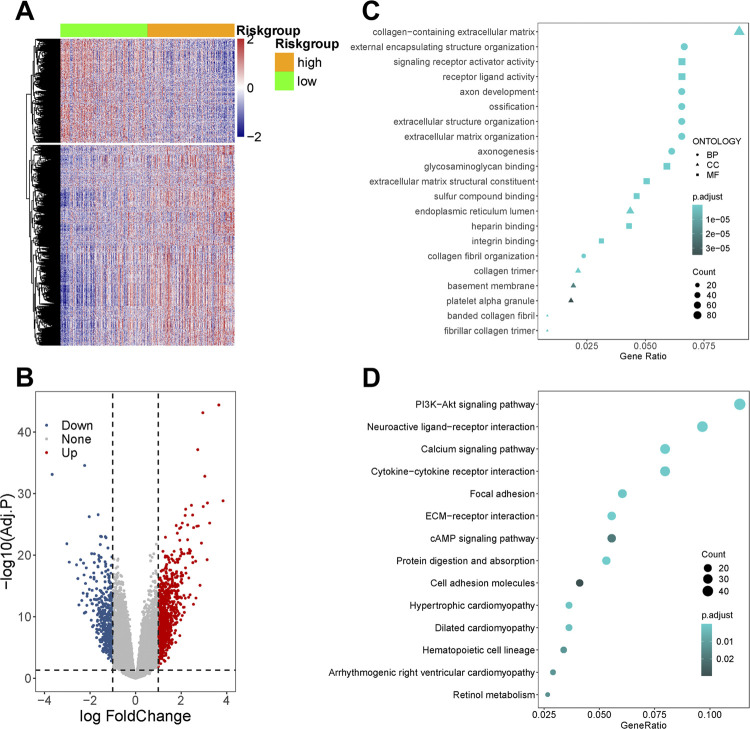
Functional enrichment and pathway analysis.

One thousand two hundred ninety-nine DEGs between high-risk group and low-risk group were defined, including 847 up-regulated genes and 452 down-regulated genes (Figure 7 A and B). The molecular characteristics and pathways of risk score were investigated by enrichment analysis. For biology process (BP), cell component (CC), and molecular function (MF) categories in GO analysis, the top 7 significantly enriched by risk score in each category were displayed in dot plot (Figure-7C). For KEGG analysis, 14 pathways were significantly enriched (FDR < 0.05; Figure-7D). Those terms were principally concentrated in extracellular matrix structural constituent, fibrillar collagen organization, cell-cell adhesion, signaling transmission pathway of PI3K and Calcium, which were consistent with the results of GSEA (Table-S5). (Click here). In oncogenic signatures, angiogenesis factors (VEGF, PDGF, and PGF), mTOR signaling pathway, E2F1 transcriptional factor, polycomb repressive complex 2 (PRC2), cAMP, and KRAS were enriched by high-risk score, while p53 was downregulated. Implying the close association of gene-based risk score with the occurrence, development, and metastasis of tumor.

### Correlation between risk score and tumor immune infiltrating cells (TICs)

**Figure 8:**
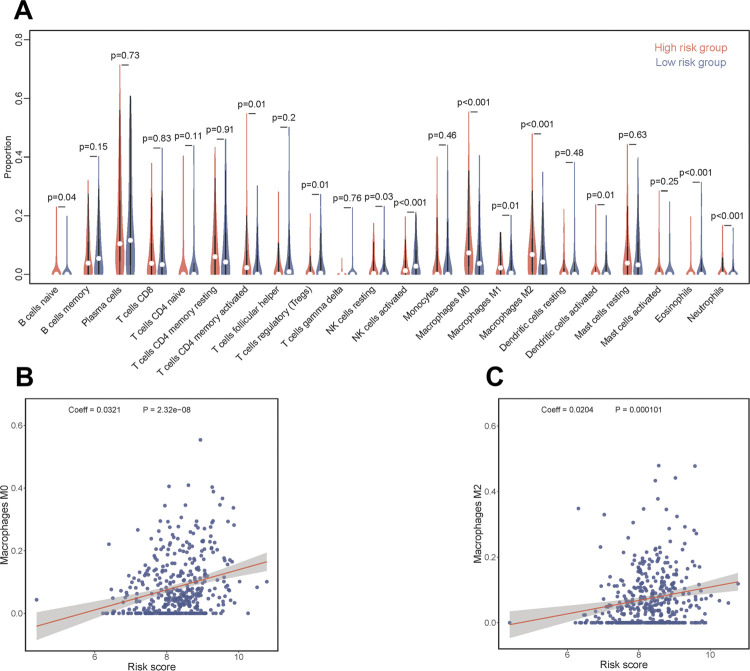
Quantification of immune cell infiltration.

The relationship between risk group and immune cells infiltration were analyzed using CIBERSORTx database. Results presented the proportion of macrophages M0 (p < 0.001) and M2 (p < 0.001) were elevated in high-risk group (Figure-8A). Linear regression further revealed that the immune infiltration levels of macrophages M0 (Coeff = 0.032, p < 0.001) and M2 (Coeff = 0.020, p < 0.001) were positively correlated with risk scores (Figures 8 B and C).

## DISCUSSION

BC is a biologically diverse disease and progresses to multiple clinical outcomes. Especially for MIBC, which is more aggressive, with a higher acquisition of genomic instability and mutation rate (37). The present grading and staging system for BC mainly depends on radiography or pathology, which evaluates the prognosis by tumor infiltration depth instead of biological characteristics. This is insufficient for predicting tumor progression, migration, and therapeutic sensitivity (38). Among variety of medicines, Platinum-based chemotherapy is the first-line treatment in systematic treatment for MIBC (39). But, platinum resistance remains a challenging issue. Genetic biomarkers would be viable to represent heterogeneity among BC, predicting the sensitivity of urothelial neoplasms tissues toward platinum. Previous studies have reported several prognostic gene signatures that are related to tumor microenvironment, immune cell infiltrating, unfolded protein response, and so on (40–42). However, signature associated with platinum-based chemotherapy resistance has not been recorded yet. Invigored by this, we attempted to identify a series of genes that were able to discriminate the platinum-based therapeutic benefits as well as predict the outcome of BC patients.

In the current study, we combined a variety of regression analyses, ten essential genes (PPP2R2B, TSPAN7, ATAD3C, SYT15, SAPCD1, AKR1B1, TCHH, AKAP12, AGLN3, and IGF2) that participating in both platinum-based chemotherapy resistance and survival were selected, then a prognostic model enrolling these genes was established. The risk score did well in stratifying patients into different risk groups, patients in low-risk group experienced a considerable survival advantage compared to those with a higher risk score. Cox regression showed the negative correlation between risk score and OS (HR = 2.7, p < 0.0001). Based on risk score and salient clinical features, a nomogram was generated to present the survival rate graphically. The nomogram model could yield a C-index of 0.727 and an AUC value up to 0.819. DCA and calibration curves also proved its promising prognostic ability. As shown in DCA, by incorporating risk score, the predictive power of clinical characteristics was strengthened. The stability and reproducibility of risk score were examined in other three independent BC datasets including cisplatin-treated patients. Moreover, a high-risk score was significantly associated with platinum resistance in BC patients. All together indicated the potential value of our model in clinical decision about platinum sensitivity and overall survival.

Among those ten genes, TSPAN7 and IGF2 have previously been reported to participate in bladder cancer progression and might be potential therapeutic targets. TSPAN7 exerts an anti-tumor effect via the PTEN/PI3K/AKT pathway in urothelial carcinoma (43). IGF2 participates in cell survival, growth and reproducing, and is overexpressed in variety of malignancies. IGF2 could contribute to anti-tumor therapy based on its regulation, modification, and downstream signaling way. IGF2 regulates PI3K/AKT/mTOR signaling pathway, targeting on IGF2 is a new therapeutic strategy for bladder cancer, and obstructing IGF2 signaling way could make cancer cell reacquire sensitivity to Taxol. Besides, among four kinds of IGF2 promoters, IGF2-P3 and IGF2-P4 have a high expression in bladder tumor tissues compared with normal bladder, which confirming their values in target therapy (44, 45).

In addition, most genes in our model were involved in the signaling pathways regulation in other kinds of cancer. In breast cancer, the expression level of PPP2R2B is significantly correlated with a longer distant metastasis-free survival and recurrence-free survival. Downregulation of PPP2R2B reduces the effect of trastuzumab or lapatinib on mTOR signaling, thus weaken the anti-HER2 sensitivity (46). What is more, PPP2R2B also involves in polarization of macrophages, the dysregulation of PPP2R2B would promote macrophages polarizing to M2, facilitating the immune evasion (47). Although TSPAN7 is an anti-tumor factor in bladder cancer, it promoted lung cancer progression by inhibits the expression of E-cadherin and vimentin, which raises the level of N-cadherin (48). It also has an interaction with the activation of β1 integrin-mediated downstream FAK-Src-Ras-ERK1/2 signaling pathway in osteosarcoma (49), boosting the cell epithelial-mesenchymal transition (EMT) process, indicating that the mechanisms of this gene and its downstream products still need to be explored. In lung cancer, AKR1B1 is a STAT3 activator that promotes glutathione de novo synthesis, eliminates ROS, protects cell from death, and reduces EGFR TKI drug sensitivity by upregulating the cystine transporter SLC7A11, it would be a therapeutic target for dealing with EGFR TKI resistance (50). TCHH methylation might play a potential role in the induced pluripotent stem cell (iPSC) differentiation, a higher level of TCHH methylation is observed in colorectal cancer liver metastasis sites and exhibits an association with tumor volume (51). Inspired by the multiple roles of these genes in other neoplasms, we carried on an analysis to 123 cisplatin-treated metastatic gastric cancer patients (GSE14208) used our risk score model, low-risk group exhibited better clinical outcomes than high-risk group not only in overall survival but also in progression-free survival, proving the robustness of our prognostic model.

To delineate the molecular mechanisms underlying the risk score, we executed enrichment analyses. GO and KEGG revealed that the risk score might play its role in extracellular matrix remodeling and cell-cell adhesion, which play an important role in tumor progression and metastasis. GSEA confirmed a significantly reduced p53, together with an elevated level of angiogenesis, histone methylation, silk/threonine kinase, cAMP, and K-ras gene in high-risk group. Many studies have stressed the role of these terms in chemotherapy resistance. For example, RAS and p53 promote or inhibit cisplatin resistance via regulate cellular apoptosis and autophagy in the opposite direction (52). Furthermore, the high-risk score was significantly associated with increased tumor infiltration of macrophages. Tumor-associated macrophages (TAMs) are crucial part of tumor immune microenvironment (TIME). Macrophages would be polarized to two phenotypes (M1 and M2), M1 suppresses tumor while M2 boosts tumor development. Exosomes derived from M2 have been shown to contribute to cisplatin resistance (53), and a repolarization of M2 to M1 would be a strategy to restore sensitivity toward platinum (54). These results denoted the reliability association between risk score and platinum sensitivity. Our model might have a practical value for prognosis stratification and early determination of therapeutic benefits.

However, our study also has some limitations. First, on account of the insufficiency of data resources, the platinum resistance-related DEGs were picked from one RNA-seq dataset (TCGA-BLCA dataset), it would be better if we could integrate transcriptomic data from more datasets. Second, we only know those patients had undergone platinum-based chemotherapy, but the exactly therapeutic strategies, such as GC or ddMVAC, were inaccessible to us. The mechanisms of these resistance might contain other than platinum therapy. Third, other information like blood and urine composition analyses, dietary habits, and lifestyle, is unclear. Forth, the proportion of patients that have undergone platinum-based chemotherapy and have definitive records of therapeutic response is relatively small, the inclusion of more eligible patients would be helpful to enhance reliability of our results. Fifth, as all conclusions in this study were processed through bioinformatics, additional biological experiments and multicenter clinical trials will assist in investigating the function of the 10 genes, as well as testing the prognostic ability in the actual world. Despite above limitations, this is the first prognostic model derived from platinum resistance and tumorigenesis, which could transform gene expression matrix into risk score and powerfully stratify patients into different prognostic groups. Bringing age and TMN stages into our model would further boost the predictive capacity. What is more, the clinical risk judgment based on an objective score system accompanied by a nomogram could also reduce the deviation arising from subjective factors of observers. All above demonstrated that our gene-based risk score model has satisfying potential of predicting platinum therapeutic effect and it could assist in conducting personalized treatment.

## CONCLUSIONS

In summary, we identified a gene-based risk score model for bladder cancer patients that has clinical prognostic value not only in survival but also in platinum-based chemotherapy sensitivity. This finding has reference value to clinical treatment decisions and deepens our understanding of platinum-based chemotherapy resistance.

## Figures and Tables

**Figure S1:**
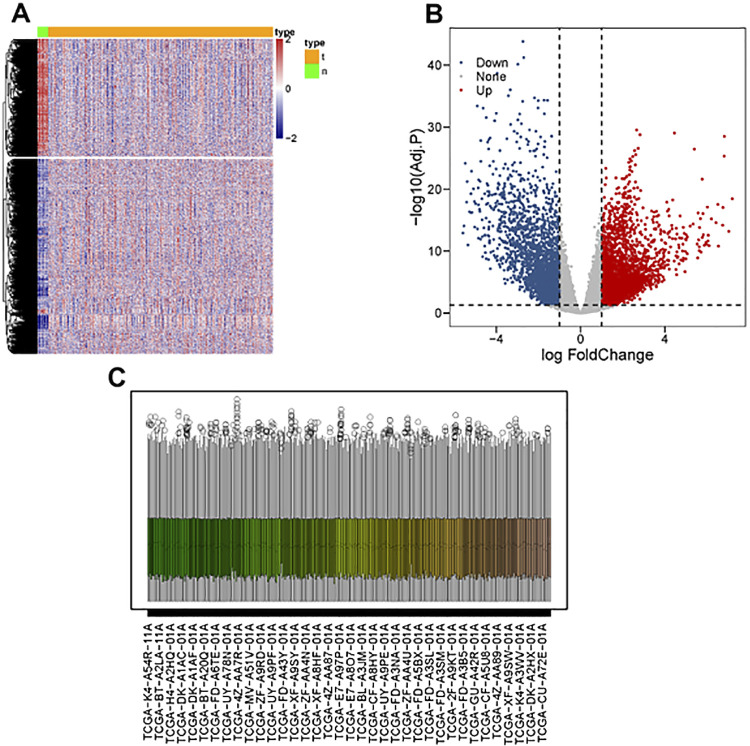
Differentially expressed genes of TCGA-BLCA cohort.

**Figure S2:**
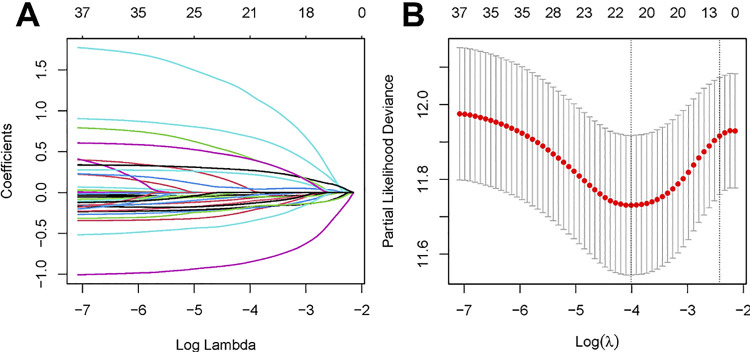
The LASSO regression.

**Figure S3:**
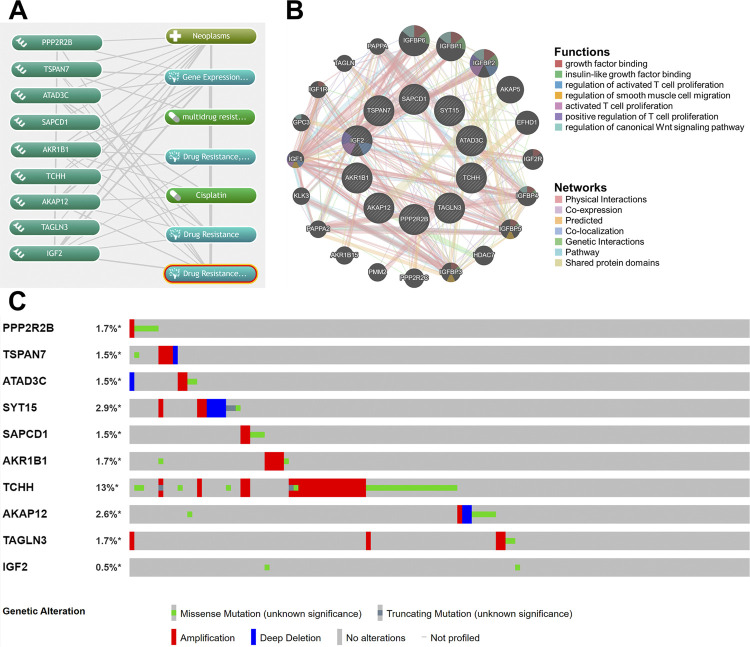
Forest plot of 10 genes in prognostic model.

**Figure S4:**
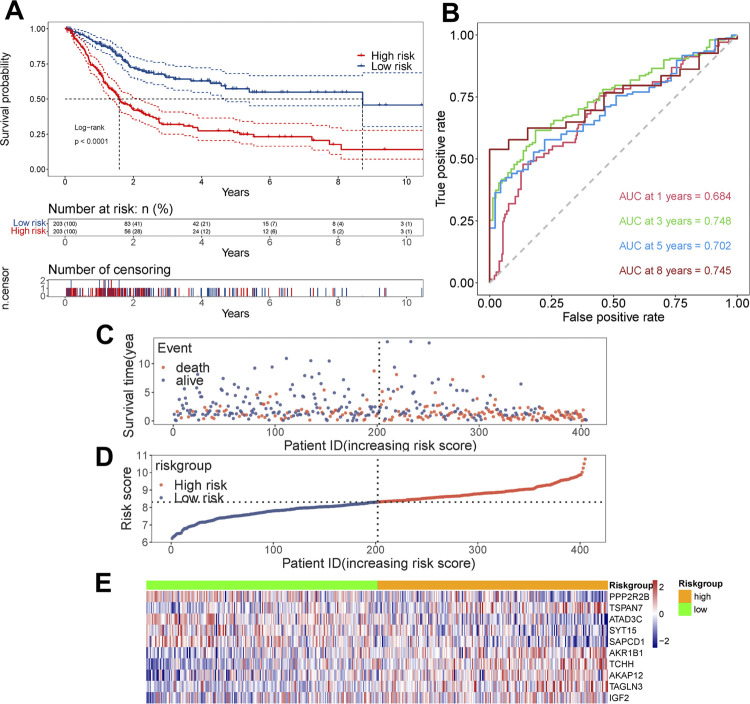
Expression levels of genes in bladder tumor and normal bladder tissues.

**Figure S5:**
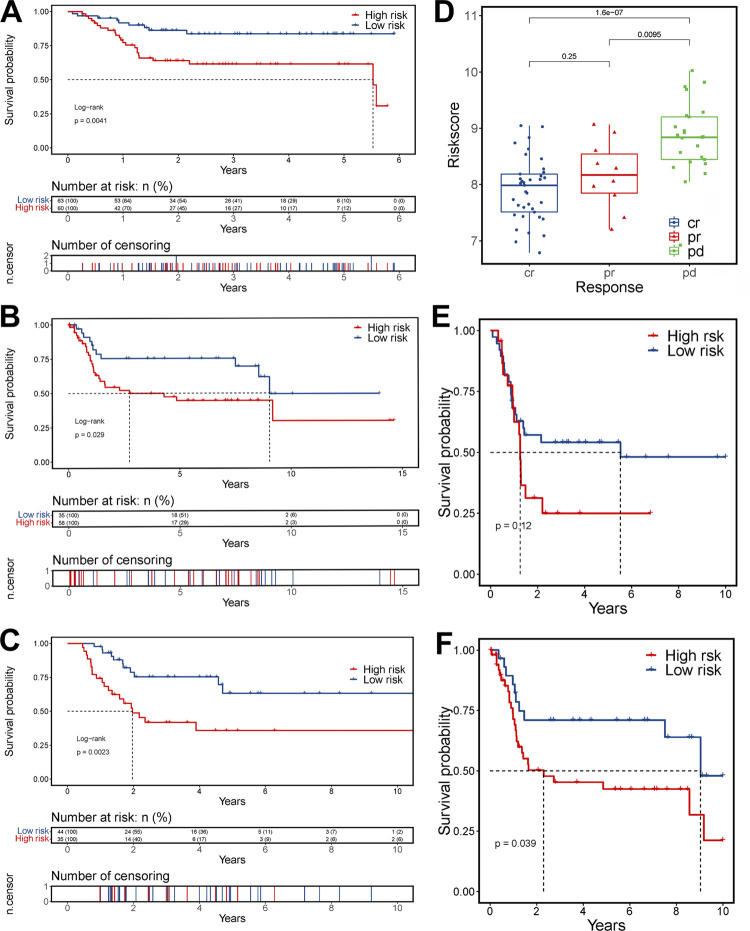
Subgroup analysis of risk group in training dataset.

**Figure S6:**
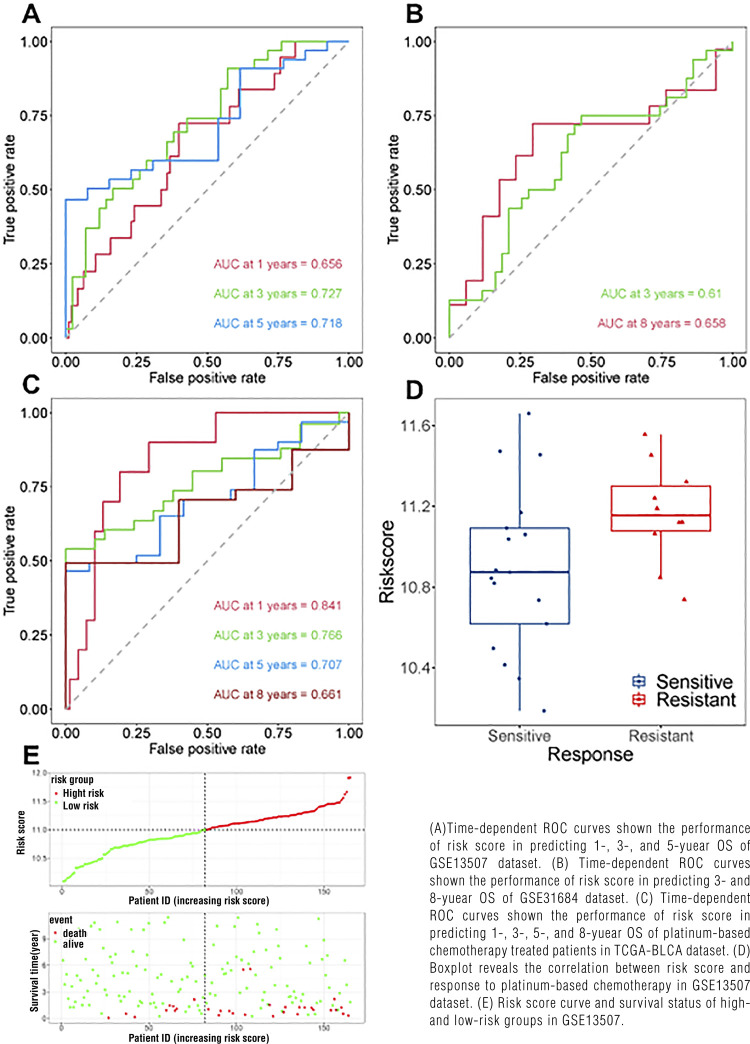
Evaluating prognostic performance of gene-based model in validation group.

**Figure S7:**
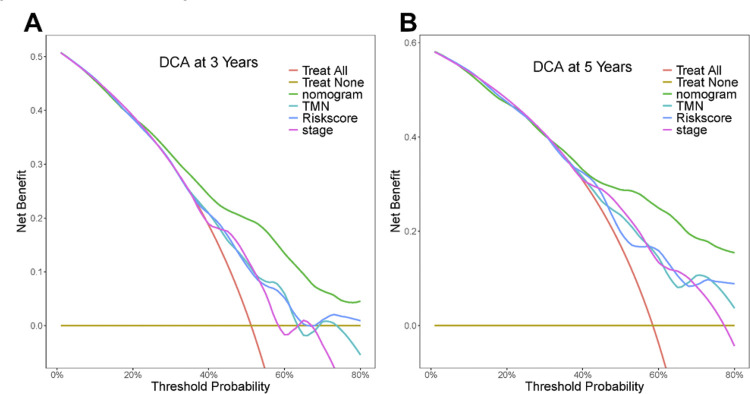
Estimation of nomogram.

## ABBREVIATIONS

BC: bladder cancer
BLCA: bladder urothelial carcinoma
MIBC: muscular invasive bladder cancer
NMIBC: non- muscular invasive bladder cancer
TCGA: The Cancer Genome Atlas
GEO: Gene Expression Omnibus
DEGs: differentially expressed genes
OS: overall survival
RFS: recurrence-free survival
PFS: progression-free survival
LASSO: least absolute shrinkage and selection operator
AIC: Akaike's information criterion
HR: hazard ratio
VIF: variation inflation factors
ROC: receiver operating characteristic
AUC: area under the curve
C: index: concordance index
DCA: Decision curve analysis
GO: Gene Ontology
KEGG: Kyoto Encyclopedia of Genes and Genomes
FDR: false discovery rate
GSEA: gene set enrichment analysis
THPA: The Human Protein Atlas
IHC: immunohistochemistry
TMN: The pathological T, M and N stages of tumor punctuation
